# Comorbidity Cohort (2C) study: Cardiovascular disease severity and comorbid osteoarthritis in primary care

**DOI:** 10.1186/1472-6963-12-295

**Published:** 2012-09-03

**Authors:** James A Prior, Claire A Rushton, Kelvin P Jordan, Umesh T Kadam

**Affiliations:** 1 Health Services Research Unit, Innovation Centre 1, Keele University Science & Business Park, Keele University, Staffordshire ST5 5NB, UK; 2 Arthritis Research UK Primary Care Centre, Primary Care Sciences, Keele University, Staffordshire ST5 5BG, UK

**Keywords:** Comorbidity, Cardiovascular diseases, Osteoarthritis, General practice, Cohort studies

## Abstract

**Background:**

Two of the commonest chronic diseases experienced by older people in the general population are cardiovascular diseases and osteoarthritis. These conditions also commonly co-occur, which is only partly explained by age. Yet, there have been few studies investigating specific a priori hypotheses in testing the comorbid interaction between two chronic diseases and related health and healthcare outcomes. It is also unknown whether the stage or severity of the chronic disease influences the comorbidity impact. The overall plan is to investigate the interaction between cardiovascular severity groups (hypertension, ischaemic heart disease and heart failure) and osteoarthritis comorbidity, and their longitudinal impact on health and healthcare outcomes relative to either condition alone.

**Methods:**

From ten general practices participating in a research network, adults aged 40 years and over were sampled to construct eight exclusive cohort groups (n = 9,676). Baseline groups were defined on the basis of computer clinical diagnostic data in a 3-year time-period (between 2006 and 2009) as: (i) without cardiovascular disease or osteoarthritis (reference group), (ii) index cardiovascular disease groups (hypertension, ischaemic heart disease and heart failure) without osteoarthritis, (iii) index osteoarthritis group without cardiovascular disease, and (vi) index cardiovascular disease groups comorbid with osteoarthritis. There were three main phases to longitudinal follow-up. The first (*survey population*) was to invite cohorts to complete a baseline postal health questionnaire, with 10 monthly brief interval health questionnaires, and a final 12-month follow-up questionnaire. The second phase (*linkage population*) was to link the collected survey data to patient clinical records with consent for the 3-year time-period before baseline, during the 12-month survey period and the 12 months after final questionnaire (total 5 years). The third phase (*denominator population*) was to construct an anonymised clinical data archive for the study five year period for the total baseline cohorts, linking clinical information such as diagnosis, prescriptions and referrals.

**Discussion:**

The outcomes of the study will result in the determination of the specific interaction between cardiovascular severity and osteoarthritis comorbidity on the change and progression of physical health status in individuals and on the linked and associated clinical-decision making process in primary care.

## Background

As people get older, they are more likely to experience a chronic disease, such as cardiovascular disease (CVD) or osteoarthritis (OA), and many will experience two or more chronic diseases at the same time [1]. With increasing life spans, each individual is more likely to experience multiple chronic diseases. Yet, in the day-to-day management of patients, the focus has often been on single chronic disease [2]. Such approaches do not acknowledge or address the common experience of older populations with multiple chronic diseases such as CVD and OA. Implications for the impact on health care and health care systems lie, for example, in the variations in the clinical decision-making process as exemplified by referrals [3] and in mortality outcomes [4], which could be explained by the presence of multiple chronic diseases in the same individual. It has been argued that the management of single diseases may distort the provision of good health care by not addressing the potential interactions of different conditions and therefore not appropriately assessing the management of each chronic illness in the real clinical situation [5]. Consideration of potential patterns of care in patients with comorbidity requires a broader perspective on management and the clinical pathways, and alternative approaches are required to address this problem [6]. From an international perspective, in an ageing European population, this issue is set to become an increasing public health priority. Current estimates range up to 30 million European sufferers with two or more chronic diseases, with further increases likely as the number of older Europeans expands by an estimated 30% in the next 25 years [7].

Two of the commonest chronic diseases experienced by older people in the general population are CVD and OA [8]. Cardiovascular disease, shares many of the chronic disease characteristics shown by OA, and is an important cause of disability as well as mortality. A range of studies have shown that CVD is associated with poor physical health, and this relationship influences management progression and health care outcomes. For example, people with poor physical health are likely to report greater CVD health needs [9], the progression of CVD symptoms is likely to be associated with poor physical health [10] and this in consequence is likely to lead to higher hospital admissions and mortality [11]. In a similar pattern to OA, co-existing depression adversely influences symptoms of CVD and is more likely to be associated with poor physical health [12]. Specific studies have also shown that poor physical health is associated with CVD that range from hypertension, atrial fibrillation, angina, myocardial infarction to heart failure. Some studies indicate that poor health in hypertension is unexplained by socio-demographic factors or comorbidity [13], in atrial fibrillation is dependent on the severity of symptoms [14], in angina patients is associated with depression and anxiety [15], and in myocardial infarction or heart failure is associated with poor health care outcomes [16,17].

Osteoarthritis is the most frequent reason for restricted activity in daily life [18] and has a high impact on health care use and costs [19], both in hospital (for example, joint replacements [20]) and in primary care in relation to consultations and drug use [21]. The prevalence of many other disabling conditions also rises with age, and some common chronic conditions can be found alongside OA, including CVD [22]. We have previously shown that there are specific associations in OA sufferers in general practice [23] and that the combination of OA and comorbidity is associated with much poorer physical health [24]. Several studies have shown that OA and specific CVD are associated together and this co-occurrence is independent of age [25,26]. Explanations for this finding include pathologic links, similar and shared risk factors or intermediary links, such as drugs (anti-inflammatories). In addition to adverse mortality outcomes, previous OA studies have also shown that people with CVD comorbidity have poor quality of life, and that co-existing conditions such as depression can influence similar outcomes [27,28].

In primary care, where multiple morbidity is the rule rather than the exception [29,30], general practitioners and primary care by definition deal with many different morbidities presented by the same individual. As each encounter contributing to multiple morbidity is routinely recorded during consultations and subsequently in historical records, so a catalogue of health states emerges through which an individual passes over time. Such health events might be linked to each other [31], because they represent overlapping syndromes [32] or are a result of shared causes or mechanisms, and their interactions might help to explain different patterns in health course or progression. Studies of multiple morbidity in primary care, based on a limited number of empirically selected chronic conditions, have shown that it is negatively associated with overall health [33] and that it is associated with increased referral to secondary care and increased health care costs [1,34]. Whilst, studies of the association between specific chronic diseases and overall health have been completed, especially in relation to changes within intervention studies, very few studies have examined the patterns of change in health that leads to consultation [35] and in those with specific dual chronic diseases at the same time. How comorbidity influences short and longer-term health status in CVD or OA, how it causes changes in health status, and how it influences health care management decisions is unknown.

Within the broad terms of CVD or OA, there will be spectrum of different disease categories for each individual chronic disease. So for example, the term ‘CVD’ encapsulates a spectrum ranging from hypertension to chronic congestive heart failure as outlined, and ‘OA’ encapsulates a spectrum of joint-specific problems. In each spectrum, each stage implicitly carries the notion of the process of disease severity related to a specific outcome. For example, in people with OA, impact on mobility will be dependent on the joint site and whether there is pain with or without radiographic change, whereas, the stage of CVD will determine outcomes, such as health status and mortality. In the course of chronic disease development in populations, it is the stages within each disease process that offers one definition of ‘severity’. Studies, for example, in the CVD field, suggest that the lifetime risk of different CVD varies with age and the related risk factors [36-38]. So instead of simply using broad disease categories, the spectrum of CVD ‘severity’ potentially offers an empirical way of exploring the disease gradient to investigate whether the interaction between two individual chronic diseases and its impact on health is over and above that which we might expect from simply combining the individual effects.

Using an empirically defined order of disease severity we intend to use hypertension, ischaemic heart disease (angina or myocardial infarction) and heart failure as indicators of CVD severity with comorbid OA defined as a single broad category. In this study we propose to investigate the specific interaction of CVD severity and OA comorbidity on:

(i) the progression of physical health (with the null hypothesis: that the adverse influence on physical health is the same for CVD and comorbid OA compared to those with either index condition alone), and

(ii) the associated clinical decisions in consulting adult general practice populations aged 40 years and over compared to consulters with either condition alone or without either condition (with the null hypothesis: that clinical decisions are the same for CVD and comorbid OA compared to those with either index chronic disease).

## Methods

### Setting

Our study will be carried out in ten general practices, from North Staffordshire, Stoke-on-Trent and Cheshire. These practices are part of a local research network, the Primary Care Musculoskeletal Research Consortium. These practices, supported by the Primary Care Research West Midlands North (PCR WMN) network, cover a wide range of socio-economic groups and includes practices that have actively participated in routine collection of clinical data using computer records for the purposes of epidemiological study. Clinical information relating to all morbidity and drug therapies is recorded using standard classifications of Read codes [39] and BNF (British National Formulary) respectively [40]. Ethics permission was sought and given by the Cheshire Research Ethics Committee (REC ref no: 09/H1017/40).

### Study population

The cohort study is based on the recruitment of four main groups aged 40 years and over who have either a record or consultation for: (i) No CVD or OA (reference); (ii) CVD without OA; (iii) OA without CVD and (iv) CVD and OA (comorbid cohort). The sampling of these groups from the ten general practices was in a three-year period beginning November 2006 and ending January 2010.

### Identifying cohort samples

*CVD cohorts*: Using the CVD registers and historical information, all adults aged 40 years and over who had a record (main Read code Chapter G: “Cardiovascular system diseases”) and on active registration at the end of the 3-year study time-period will be identified. Cohorts will then be based on the three CVD severity categories of hypertension (Read and daughter codes beginning with G20), ischaemic heart disease (Read and daughter codes beginning with G3.) and heart failure (Read and daughter codes beginning with G58 and heart failure codes related New York Heart Association (NYHA) classification), as these conditions are currently part of the national Quality Framework of clinical recording, a means by which General Practitioners (GP) are reimbursed as part of chronic disease management. The CVD groups will be organised into three exclusive severity groups, which means that allocation to a cohort group will be based on the most severe category e.g. if an individual had consulted for hypertension and heart failure, they would be in the heart failure cohort. Previous studies have indicated that the recording quality of such information is likely to be high [41,42]. These three cohorts will be separated into two sub-groups of (i) patients who had not consulted for OA in the same time period (index CVD cohort) and (ii) patients who had also consulted for OA in the same time period (comorbid cohort). All those in the three CVD severity groups who had comorbid OA were sampled, as were those with heart failure or ischaemic heart disease but no OA. However, those with hypertension but without OA were randomly sampled (stratified by practice) given the high prevalence of this group (see Sample size).

*OA cohort*: Using the general practice records for the same time period (2006–2010), all adults aged 40 years and over, who are actively registered and who either have a historical record or consultation for OA (Read and daughter codes beginning with N05, and codes related to OA joint replacement (7 K2 or 7 K3)) will be identified. This cohort group will be exclusive of those patients who had also consulted for any CVD-related diagnosis (as stated above) in the same time period.

*Non-index cohort*: Using the general practice records for the same time period (2006–2010), a random sample stratified by practice of all adults aged 40 years and over without a historical record or consultation for the study specific CVD and OA codes will be identified. This sample will provide a random reference group for the other main cohort categories.

### Data collection

There are 3 phases to the study data collection. The first phase (*survey population*) will use postal questionnaires to obtain self-reported health information at baseline, monthly interval and 12-month follow-up. The second phase (*linkage population*) will link the survey data to consultation data, for patients who will give written consent to access their clinical records from general practice in the baseline questionnaire. The third phase (*denominator population*) will construct an anonymised database for the whole cohort for the total five-year time-period. So the denominator cohort constitutes the whole sample from which the survey sample will be drawn. The denominator sample will provide the basis for a distinct cohort sample in its own right, as well as addressing selection comparisons between people who took part in the survey and those who did not, and people who gave consent to record review and who did not.

### Phase 1: Survey population

**Figure 1:**
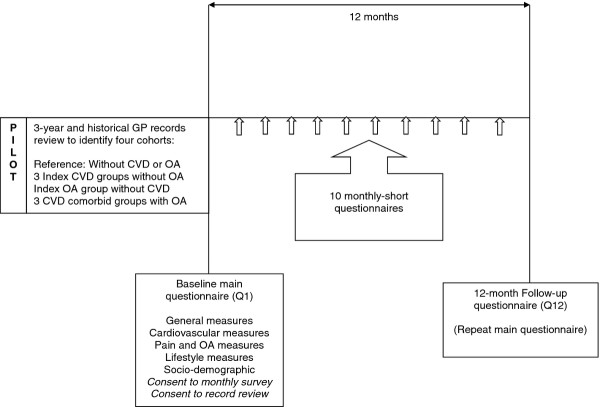
2C study design.

Overall, study participants will be invited to complete 12 questionnaires – baseline and 12-month follow up, and between these time-points, 10 monthly short-interval questionnaires (Figure 1). A pilot study in one practice on 500 patients was carried out to test the feasibility of sampling and testing of the questionnaire methods.

### Baseline survey

The identified sampled cohorts will be sent a baseline questionnaire (Q1), which will include generic and specific-health measures (Table 1). Measures have been identified on the basis of the primary focus on CVD and OA diseases and other specific measures selected on the basis of conceptual links between the disease and outcome of interest in the follow-up phase.

**Table 1:** Comorbidity Cohort (2C) study measures

		**Survey measures**	
**Data source**	**Factors**	**Baseline & 12-month follow-up**	Short monthly**
Clinical records	8 disease cohorts	· Read code classification*	
	Consultation comorbidity	· Kadam severity classification	
	Blood tests & investigations	· Read code classification*	
	Drugs prescribed	· British National Formulary (BNF)	
	Referrals	· Read code classification*	
Survey data	General measures	· Short form 12 (SF-12) health survey	· SF-12
		· Short form 36 (SF-36) health survey – Q2 only	· SF-36, Q2
		· EuroQol (EQ-5D)	
		· Hospital Anxiety and Depression (HAD) scale	
		· Medical Outcomes Study (MOS) - Sleep Scale	· Qs 1 & 2 only
		· Functional Assessment of Chronic Illness (FACIT) –	· Qs 5 & 6 only
		Fatigue	
		· Brief Illness Perception Questionnaire (B-IPQ)	
		· Social Networks	
	Cardiovascular measures	· Seattle Angina Questionnaire (SAQ), UK version	· Qs 2 & 3 only
		· Kansas City Cardiomyopathy Questionnaire (KCCQ)	· Qs 3 & 7 only
		· Rose Angina Questionnaire	
		· Palpitations (based on [50])	· Q1 only
		· Vertigo Severity Scale (VSS)	· Q1 only
	Pain and OA measures	· Pain manikin, pain frequency & pain intensity	· Pain frequency
		· Knee Injury & Osteoarthritis Outcome Score (KOOS) –	
		Physical Function Shortform	
		· Hip Injury & Osteoarthritis Outcome Score (HOOS) –	
		Physical Function Shortform	
	Lifestyle	· Body Mass Index (BMI), Alcohol, Smoking	
		· Short Questionnaire to Assess Health Enhancing	· Qs 2a, 2c, 2e only
		Physical Activity (SQUASH)	
	Other	· Eyesight/hearing	
		· Body shape	
Survey & clinical records	Socio-demographic	· Age, Gender, Deprivation	

*Read code classification is a standard clinical coding system used in British general practice; **question numbers refer to the original questionnaire.

For the *primary outcome* of interest, we will use the Short-Form-12 (SF-12) (version 2) health survey as a generic measure of physical health, specifically the Physical Component Summary (PCS) score [43]. We will also include an assessment of physical activity [44] and Hospital Anxiety and Depression (HAD) questionnaire will be included as a measure of psychological status, that influences both CVD and OA [45].

The CVD-specific measures will include the: Seattle Angina Questionnaire [46], Kansas City Cardiomyopathy questionnaire [47], Rose angina questionnaire [48], and symptoms of palpitations [49] and dizziness [50].

The OA-specific measures will include the: Knee injury and Osteoarthritis Outcome Score (KOOS – physical function component) [51], Hip injury and Osteoarthritis Outcome Score (HOOS – physical function component) [52], pain scale, pain manikin [53], joint pain, and other links previously reported in chronic disease literature in relation to the symptoms of tiredness [54] and sleep [55].

Survey participants will also be requested for consent to the monthly short-interval questionnaires and for permission to the subsequent review of their clinical records. Participants will be “tagged” in their general practice registers as ‘2C’ study participants.

### Measurement of health in the 12-month follow-up period

With baseline consent, participants will be sent 10 monthly short-interval questionnaires (4 pages), including the SF-12, pain measures and CVD symptom measures. At the end of the study period, all study participants will be sent a 12-month follow-up postal questionnaire (Q12) using the same measures as used in the baseline survey.

### Phase 2: Survey-consultation linkage population

The linked consultation data covers a total five-year time-period, for 3 years before the baseline survey, 12 months of the survey, and the 12 months after the full follow-up survey. We will measure the clinical (GP or nurse) decision process associated with the survey cohort follow-up. Using the same study samples for both phases allows the linkage of the self-reported health status, morbidity and the decision process experienced by that individual patient in the 12-month period.

After the completion of the survey phase, the clinical record data for cohort groups will be downloaded with prior patient consent. Clinical decisions in consenting patients will be measured following the baseline survey on the basis of: (i) drug treatment changes (new, repeat, dose, type), (ii) investigations such as blood tests or X-rays, (iii) referrals (in-practice and external, investigation or second-opinion) or (iv) no change in any of these measures. Drug use will be measured on the basis of new index-related treatments (i.e. analgesia for OA and CVD-related therapies), or change in doses used for those on pre-existing drug treatments. Investigations will cover index-specific indications, cardiological (e.g. Electrocardiogram (ECG), Cholesterol) or rheumatological (e.g. X-Rays). Referrals will include those relevant to the index conditions (i.e. physiotherapists and allied therapists, rheumatologists, orthopaedics and cardiologists) and all other referrals. Other measurements for the cohorts will include all other comorbidity using the Kadam severity classification [56,57], specific morbidities such as cerebrovascular disease and peripheral arterial disease, and co-drug therapies used.

### Phase 3: Anonymised denominator population

Phase 1, survey population method is defined by the responders who take part in the study, phase 2, the survey-consultation method is subject to the consenters who will give permission for this link to occur. Therefore, phase 3 will focus on the total cohort denominator population and the anonymised clinical data within the medical records of each individual patient to allow methodological assessment of response biases in the cohort samples. Patients who explicitly stated to their general practice that they did not wish to take part in any research or share their clinical data are excluded from this phase.

**Figure 2:**
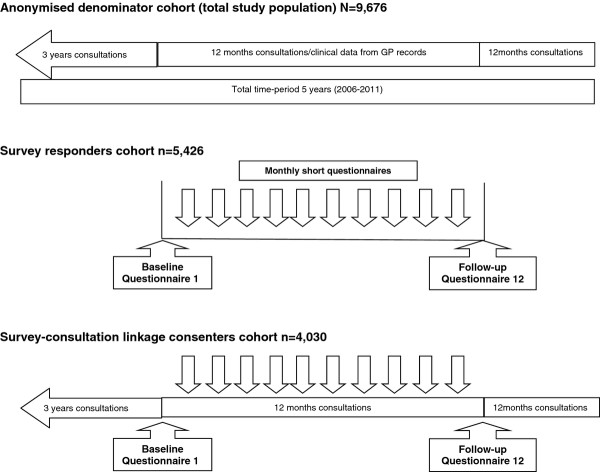
Comorbidity Cohort (2C) design.

For the total denominator population, invited to take part at baseline, anonymised clinical data with patient diagnostic information, prescribed drugs, blood tests and investigations, referrals and linked healthcare activity will be collected. This anonymised data archive covers a total five year time-period (between 2006 and 2011) from 3 years prior to the baseline survey to 12 months after the 12 month follow-up survey (Figure 2).

### Sample size

The pilot study was used to guide the sample size needed. The primary outcome of the study is the mean change in PCS score from baseline to 12-month follow-up. In order to compare the change in PCS score between the whole comorbid (OA and CVD) cohort and the OA index cohort we estimated that at least 394 patients responding at 12 months per cohort were needed (confidence level 0.05; power 80%) to detect a between cohort effect size of 0.2. Based on expected responses of 60% at baseline and 70% at 12 months, this required 939 patients to be invited with comorbid OA and CVD and 939 with OA alone. Assuming 75% consent to medical record review, this number would also give 423 people in each cohort responding at baseline and consenting to record review. However, to maximize numbers in the index CVD cohorts (hypertension, ischaemic heart disease and heart failure), OA index and comorbid groups and in order to allow comparison between the CVD severity comorbid cohorts with their related index CVD cohorts, we invited all eligible patients with these conditions to participate. The exceptions were the index hypertension cohorts and index OA cohorts where we randomly sampled patients stratified by practice due to the large size of these groups. Similarly, we also randomly sampled patients from the reference group without CVD or OA.

### Analysis

#### Phase 1: Survey population

The initial analysis will use independent t-tests to compare the OA and CVD comorbid cohorts with mean change in PCS over the 12-month period. We will then examine the influence of CVD severity, comorbidity and baseline covariates on mean change in PCS score using multiple linear regression methods, including cohort group, anxiety and depression, Body Mass Index (BMI), and socio-demographic characteristics (i.e. age, gender, neighborhood level deprivation [58]) as explanatory variables. These analyses will be repeated with change in the Mental Summary Component (MCS) score of the SF-12 as the outcome.

The monthly PCS scores from the SF-12 will be used as time-dependent repeated outcomes in the 12-month follow up period, to determine the association of baseline measures and time on monthly PCS score. This analysis will use repeated measures multilevel modelling, with a 2-level model (repeated PCS score within patients) with the same explanatory variables as for the 12-month analysis.

### Phase 2: Survey-consultation linkage

We will also determine cumulative onset of disability defined on the basis of the generic and disease-specific health measures. The rate of progression to severe disability in the comorbid groups will be estimated compared to the other groups using Cox regression. Attributable fractions for health status and clinical decisions (referrals and drug use), and other variables (age, gender, deprivation, obesity, consultation comorbidity) for onset or progression of disability will be determined. We will also assess time to change in clinical decisions.

### Phase 3: Denominator population

We will compare consultation patterns and management for the anonymised denominator cohort to the survey-consultation cohort to assess generalisability of our findings and estimate the likely extent of response bias.

### Preliminary data

Initial data collection has already been completed and the following data is presented to follow the sequence of sampling stages in an epidemiological cohort study (Figure 2).

### Denominator population

In the denominator cohort population, there were 9,676 people aged 40 years and over (Table 2) who were identified as baseline participants. The denominator study groups were as follows: 2,535 (26%) without CVD or OA; CVD *index* groups without OA – 1,322 (14%) with hypertension, 2,036 (21%) with ischaemic heart disease, 259 (3%) with heart failure; 1,317 (13%) in OA *index* group without CVD, and CVD groups with comorbid OA – 1,644 (17%) with hypertension, 490 (5%) with ischaemic heart disease, 73 with heart failure (1%).

**Table 2:** 2C baseline cohorts

**Disease cohort**	**Cohort ID**	**Denominator cohort N = 9676 n**	Responders cohort n = 5426 n* (%)	Non-responders cohort n = 4250 n* (%)	Linkage consenters cohort n = 4030 n** (%)
-CVD -OA	0	2535	1165 (45.9)	1370 (54.1)	820 (70.4)
+Hyp -OA	1	1322	720 (54.5)	602 (45.5)	525 (72.9)
+IHD -OA	2	2036	1196 (58.7)	840 (41.3)	915 (76.5)
+HF -OA	3	259	149 (57.5)	110 (42.5)	108 (72.5)
-CVD + OA	4	1317	828 (62.9)	489 (37.1)	617 (74.5)
+Hyp + OA	5	1644	1017 (61.9)	627 (38.1)	773 (76.0)
+IHD + OA	6	490	305 (62.2)	185 (37.8)	237 (77.8)
+HF + OA	7	73	46 (63.0)	27 (37.0)	35 (76.1)

CVD = Cardiovascular disease, OA = Osteoarthritis, Hyp = Hypertension, IHD = Ischaemic heart disease, HF = Heart failure.

Disease categories (defined by study specific Read codes in the 3 years prior to baseline survey.

-CVD -OA = no record of CVD (hypertension, IHD or HF) condition or OA; -CVD + OA = record of OA but not CVD (hypertension, IHD or HF).

+Hyp -OA = record of hypertension without OA; +IHD -OA = record of IHD without OA; +HF -OA = record of HF without OA.

+Hyp + OA = record of hypertension and OA; +IHD + OA = record of IHD and OA; +HF + OA = record of HF and OA.

*percentage of denominator population; **percentage of responder population.

*Reference cohort:* The reference cohort without CVD or OA (Table 3) was composed of the younger population, and around 69% were aged 59 years or younger. It had an equivalent number of men and women, and around 20% in the top or bottom tertiles of deprivation.

**Table 3:** Socio-demographic characteristics of the 2C study denominator population (n = 9,676)

		**Disease cohorts**							
		**Reference**	**Index CVD cohorts**			**Index OA cohort**	**CVD comorbid cohorts**		
**Factors**		**-CVD -OA (0) n = 2535 (%)**	**+Hyp -OA (1) n = 1322 (%)**	**+IHD -OA (2) n = 2036 (%)**	**+HF -OA (3) n = 259 (%)**	**-CVD + OA (4)n = 1317 (%)**	**+Hyp + OA (5) n = 1644 (%)**	**+IHD + OA (6) n = 490 (%)**	**+HF + OA (7) n = 73 (%)**
Age group (years)	40-49	892 (35.2)	106 (8.0)	72 (3.5)	5 (1.9)	138 (10.4)	33 (2.0)	4 (0.8)	0 (0)
	50-59	848 (33.5)	291 (22.0)	275 (13.5)	22 (8.5)	309 (23.5)	172 (10.4)	22 (4.5)	1 (1.4)
	60-69	513 (20.2)	416 (31.5)	617 (30.3)	54 (20.8)	445 (33.8)	499 (30.4)	106 (21.7)	4 (5.5)
	70-79	195 (7.7)	329 (24.9)	677 (33.3)	82 (31.7)	278 (21.1)	554 (33.7)	203 (41.4)	23 (31.5)
	80≥	87 (3.4)	180 (13.6)	395 (19.4)	96 (37.1)	147 (11.2)	386 (23.5)	155 (31.6)	45 (61.6)
Gender	Men	1245 (49.1)	609 (46.1)	1334 (65.5)	142 (54.8)	563 (42.7)	611 (37.2)	205 (41.8)	31 (42.5)
	Women	1290 (50.9)	713 (53.9)	702 (34.5)	117 (45.2)	754 (57.3)	1033 (62.8)	285 (58.2)	42 (57.5)
Deprivation Status*	Category 0 (most affluent)	525 (20.8)	266 (20.2)	368 (18.1)	38 (14.7)	267 (20.3)	327 (20.0)	86 (17.7)	13 (18.3)
	Category 1	1521 (60.1)	786 (59.8)	1251 (61.6)	156 (60.2)	808 (61.6)	980 (60.0)	310 (63.9)	43 (60.6)
	Category 2 (most deprived)	484 (19.1)	263 (20.0)	411 (20.3)	65 (25.1)	237 (18.1)	327 (20.0)	89 (18.4)	15 (21.1)

CVD = Cardiovascular disease, OA = Osteoarthritis, Hyp = Hypertension, IHD = Ischaemic heart disease, HF = Heart failure. *Deprivation status is based on Index of Multiple Deprivation score and available for 9,636 patients and ‘most affluent’ as the top 20% of the sample and ‘most deprived’ as the bottom 20% of the sample.

*Index CVD cohorts without OA:* The youngest age groups were in the hypertension cohort (30% aged 59 years and younger), and the oldest groups were in the heart failure cohort (69% aged 70 years and over) (Table 3). There were more men than women in both the IHD and heart failure cohorts, and a quarter of the heart failure cohort had the most deprived status.

*Index OA cohort without CVD:* Most of this cohort was aged between 50 and 80 years of age, and there were more women than men (Table 3). The age figures were in contrast to the index CVD cohorts who had a higher number in the age group 70 years and over, but the deprivation figures were similar.

*Comorbid CVD & OA cohort:* The comorbid groups were relatively older than the index groups and the age proportion for the group 70 years and over were: 57% in the hypertension comorbid group, 73% in the IHD comorbid group and 93% in the heart failure comorbid group (Table 3). There were more women than men with CVD and comorbid OA for all three groups, and IHD comorbid group had relatively fewer numbers in the top and bottom deprivation tertiles.

### Survey participation and consent

In the baseline survey population, there were 5,426 (56%) people aged 40 years and over who responded to the questionnaire. The lowest response was in the reference cohort (46%) and the highest was in the comorbid heart failure cohort (63%). The proportion of responders in the index OA cohort and the CVD comorbid cohorts were higher than the index CVD cohorts (Table 2).

The survey responders were broadly similar in terms of age, gender and deprivation characteristics, compared to the denominator population (Table 3 and 4). When comparing the responder and non-responder groups (Tables 4 and 5), non-responders were likely to be the younger groups, women and those people with the most deprived status. In the reference cohort, 74% of the non-responder group were 59 years or younger. The age proportions of the index hypertension and index OA cohort were similar, but the proportions of age groups 70 years and over were higher in index IHD (47%) and heart failure (61%) cohorts, and highest in the IHD (70%) and heart failure (93%) comorbid cohorts. Apart from the reference cohort and the index IHD cohort, there were more women than men non-responders, and around a quarter of the most deprived groups were non-responders in all study cohorts.

**Table 4:** Socio-demographic characteristics of baseline responders stratified by study groups (n = 5,426)

		**Disease cohorts**							
		**Reference**	**Index CVD cohorts**			**Index OA cohort**	**CVD comorbid cohorts**		
**Factors**		**-CVD -OA (0) n = 1165 (%)**	**+Hyp -OA (1) n = 720 (%)**	**+IHD -OA (2) n = 1196 (%)**	**+HF -OA (3) n = 149 (%)**	**-CVD + OA (4) n = 828 (%)**	**+Hyp + OA (5) n = 1017 (%)**	**+IHD + OA (6) n = 305 (%)**	**+HF + OA (7) n = 46 (%)**
Age group (years)	40-49	313 (26.8)	33 (4.6)	21 (1.7)	3 (2.0)	59 (7.1)	9 (0.9)	1 (0.3)	0 (0)
	50-59	413 (35.5)	132 (18.3)	127 (10.6)	9 (6.1)	180 (21.7)	92 (9.0)	11 (3.6)	0 (0)
	60-69	282 (24.2)	239 (33.2)	369 (30.9)	26 (17.4)	312 (37.7)	318 (31.3)	64 (21.0)	3 (6.5)
	70-79	119 (10.2)	219 (30.4)	446 (37.3)	53 (35.6)	180 (21.8)	369 (36.3)	136 (44.6)	16 (34.8)
	80≥	38 (3.3)	97 (13.5)	233 (19.5)	58 (38.9)	97 (11.7)	229 (22.5)	93 (30.5)	27 (58.7)
Gender	Men	547 (47.0)	324 (45.0)	807 (67.5)	89 (59.7)	332 (40.1)	391 (38.4)	140 (45.9)	23 (50.0)
	Women	618 (53.0)	395 (55.0)	389 (32.5)	60 (40.3)	496 (59.9)	626 (61.6)	165 (54.1)	23 (50.0)
Deprivation Status*	Category 0 (most affluent)	279 (24.0)	155 (21.7)	243 (20.4)	23 (15.4)	168 (20.4)	222 (22.0)	59 (19.6)	8 (18.2)
	Category 1	712 (61.2)	425 (59.4)	732 (61.4)	90 (60.4)	521 (63.2)	605 (59.9)	190 (63.1)	27 (61.3)
	Category 2 (most deprived)	173 (14.8)	135 (18.9)	217 (18.2)	36 (24.2)	135 (16.4)	183 (18.1)	52 (17.3)	9 (20.5)

CVD = Cardiovascular disease, OA = Osteoarthritis, Hyp = Hypertension, IHD = Ischaemic heart disease, HF = Heart failure. *Deprivation status is based on Index of Multiple Deprivation score and available for 5,399 patients and ‘most affluent’ as the top 20% of the sample and ‘most deprived’ as the bottom 20% of the sample.

**Table 5:** Socio-demographic characteristics of baseline non-responders stratified by study groups (n = 4,250)

		**Disease cohorts (Cohort ID)**							
		**Reference**	**Index CVD groups**			**Index OA Groups**	**CVD comorbid groups**		
**Factors**		**-CVD -OA (0) n = 1370 (%)**	**+Hyp -OA (1) n = 602 (%)**	**+IHD -OA (2) n = 840 (%)**	**+HF -OA (3) n = 110 (%)**	**-CVD + OA (4) n = 489 (%)**	**+Hyp + OA (5) n = 627 (%)**	**+IHD + OA (6) n = 185 (%)**	**+HF + OA 7) n = 27 (%)**
Age group (years)	40-49	579 (42.2)	73 (12.1)	51 (6.1)	2 (1.8)	79 (16.2)	24 (3.8)	3 (1.6)	0 (0)
	50-59	435 (31.8)	159 (26.4)	148 (17.6)	13 (11.8)	129 (26.4)	80 (12.8)	11 (5.9)	1 (3.7)
	60-69	231 (16.9)	177 (29.4)	248 (29.5)	28 (25.5)	133 (27.2)	181 (2.9)	42 (22.7)	1 (3.7)
	70-79	76 (5.5)	110 (18.3)	231 (27.5)	29 (26.4)	98 (20.0)	185 (29.5)	68 (36.8)	7 (25.9)
	80≥	49 (3.6)	83 (13.8)	162 (19.3)	38 (34.5)	50 (10.2)	157 (25.0)	61 (33.0)	18 (66.7)
Gender	Men	698 (50.9)	285 (47.3)	527 (62.7)	53 (48.2)	231 (47.2)	220 (35.1)	65 (35.1)	8 (29.6)
	Women	672 (49.1)	317 (52.7)	313 (37.3)	57 (51.8)	258 (52.8)	407 (64.9)	120 (64.9)	19 (70.4)
Deprivation Status*	Category 0 (most affluent)	246 (18.0)	111 (18.5)	125 (14.9)	15 (13.6)	99 (20.3)	105 (16.8)	27 (14.7)	5 (18.5)
	Category 1	809 (59.2)	361 (60.2)	519 (61.9)	66 (60.0)	287 (58.8)	375 (60.1)	120 (65.2)	16 (59.3)
	Category 2 (most deprived)	311 (22.8)	128 (21.3)	194 (23.2)	29 (26.4)	102 (20.9)	144 (23.1)	37 (20.1)	6 (22.2)

CVD = Cardiovascular disease, OA = Osteoarthritis, Hyp = Hypertension, IHD = Ischaemic heart disease, HF = Heart failure. *Deprivation status is based on Index of Multiple Deprivation score and available for 4,327 patients and ‘most affluent’ as the top 20% of the sample and ‘most deprived’ as the bottom 20% of the sample.

Of the survey responders, 4,030 (74%) gave permission to access and link their clinical records (Table 2), and this linked clinical data will be anonymised for the purposes of analyses. The lowest consent proportion was in the reference group (70%) and the highest in the CVD comorbid groups (76%).

## Discussion

There are very few comorbidity cohort studies [59] and this is the first study of its kind which has been constructed with a priori hypotheses, identifying two common chronic diseases (CVD and OA) in the general practice population, and testing their interaction in relation to self-reported health and health care outcomes. The innovative epidemiological design incorporates comorbid interaction, interaction as influenced by severity (in this example of CVD), and potential cohort impact both in the short-term and longer-term. The innovative methodological design also incorporates a denominator cohort, from which a survey population was sampled, allowing the assessment of selection and data issues. The linkage between survey data and consultation data provides the investigation of population symptoms and health, and their impact on short and longer-term healthcare outcomes.

## Abbreviations

2C: Comorbidity Cohort study; B-IPQ: Brief Illness Perception Questionnaire; BMI: Body Mass Index; BNF: British National Formulary; CVD: Cardiovascular Disease; ECG: Electrocardiogram; EQ-5D: EuroQol; FACIT: Functional Assessment of Chronic Illness; GP: General Practitioners; HAD: Hospital Anxiety and Depression questionnaire; HF: Heart Failure; HOOS: Hip injury and Osteoarthritis Outcome Score (physical function component); Hyp: Hypertension; IHD: Ischaemic Heart Disease; KCCQ: Kansas City Cardiomyopathy Questionnaire; KOOS: Knee injury and Osteoarthritis Outcome Score (physical function component); MCS: Mental Component Summary score; MOS: Medical Outcomes Study; OA: Osteoarthritis; PCR WMN: Primary Care Research West Midlands North network; PCS: Physical Component Summary score; REC: Research Ethics Committee; SAQ: Seattle Angina Questionnaire; SQUASH: Short Questionnaire to Assess Health Enhancing Physical Activity; SF-12: Short-Form 12; SF-36: Short-Form 36; VSS: Vertigo Severity Scale.

## Competing interests

The authors declare that they have no competing interests.

## Authors’ contributions

JAP coordinated the 2C study, was involved in its development, analysed and interpreted the data and contributed to the writing of this manuscript. CAR was involved in the writing and reviewing of this work. KPJ was involved in study design and provided statistical support throughout the course of the study. UTK conceived and designed this study, was involved with analysis and interpretation and contributed to the writing of this manuscript. All authors have contributed and approved the final version of this manuscript.

## Pre-publication history

The pre-publication history for this paper can be accessed here:

http://www.biomedcentral.com/1472-6963/12/295/prepub
